# Assessment of neuropsychiatric symptoms in dementia: toward improving accuracy

**DOI:** 10.1590/S1980-57642013DN70300003

**Published:** 2013

**Authors:** Florindo Stella

**Affiliations:** 1 UNESP - Universidade Estadual Paulista, Instituto de Biociências, Campus de Rio Claro. Pesquisador do Laboratório de Neurociências, Instituto de Psiquiatria - Faculdade de Medicina, Universidade de São Paulo.

**Keywords:** neuropsychiatric symptoms, assessment, scales, Alzheimer's disease, dementia

## Abstract

The issue of this article concerned the discussion about tools frequently used tools for assessing neuropsychiatric symptoms of patients with dementia, particularly Alzheimer's disease. The aims were to discuss the main tools for evaluating behavioral disturbances, and particularly the accuracy of the Neuropsychiatric Inventory – Clinician Rating Scale (NPI-C). The clinical approach to and diagnosis of neuropsychiatric syndromes in dementia require suitable accuracy. Advances in the recognition and early accurate diagnosis of psychopathological symptoms help guide appropriate pharmacological and non-pharmacological interventions. In addition, recommended standardized and validated measurements contribute to both scientific research and clinical practice. Emotional distress, caregiver burden, and cognitive impairment often experienced by elderly caregivers, may affect the quality of caregiver reports. The clinician rating approach helps attenuate these misinterpretations. In this scenario, the NPI-C is a promising and versatile tool for assessing neuropsychiatric syndromes in dementia, offering good accuracy and high reliability, mainly based on the diagnostic impression of the clinician. This tool can provide both strategies: a comprehensive assessment of neuropsychiatric symptoms in dementia or the investigation of specific psychopathological syndromes such as agitation, depression, anxiety, apathy, sleep disorders, and aberrant motor disorders, among others.

## Abstract (pt)

Neste artigo, comentamos sobre as escalas frequentemente utilizadas para avaliação de sintomas neuropsiquiátricos presentes em pacientes com demência, especialmente, na doença de Alzheimer. Os objetivos consistiram na discussão sobre as principais estratégias de avaliação dos distúrbios de comportamento e, particularmente, no exame da acurácia do Inventário Neuropsiquiátrico – Avaliação do Clínico (NPI-C) A investigação clínica e o diagnóstico das síndromes neuropsiquiátricas requerem uma acurácia apropriada. Avanços no reconhecimento dos sintomas e na acurácia do diagnóstico precoce das manifestações psicopatológicas contribuem para a instituição do tratamento adequado, tanto farmacológico como não-farmacológico. Além disso, instrumentos padronizados e validados são recomendados tanto para a investigação científica, quanto para a prática clínica. Estresse emocional, sobrecarga de trabalho e declínio cognitivo, não raro presente no cuidador idoso, podem afetar a qualidade da descrição dos sintomas vivenciados pelo paciente. Neste contexto, o NPI-C é uma abordagem promissora e versátil para a avaliação das síndromes neuropsiquiátricas na demência, com boa acurácia e confiabilidade, principalmente, com base na impressão diagnóstica do clínico. Esta escala permite uma avaliação global dos sintomas neuropsiquiátricos na demência ou pode ser utilizada como uma estratégia para a investigação de síndromes psicopatológicas específicas na demência, como agitação, depressão, ansiedade, apatia, distúrbios do sono ou distúrbio motor aberrante, dentre outros.

## INTRODUCTION

Neuropsychiatric disorders are a major clinical condition among patients with dementia, particularly Alzheimer's disease (AD). These disorders affect most of these patients, with a prevalence of up to 80%.^1,2^ In Brazil, some studies have identified a significant prevalence of neuropsychiatric disorders in AD, mainly agitation, aggression, irritability, depression, anxiety, apathy, behavior disorders, sleep disturbances, delusions and hallucinations.^3-5^

Recently, neuropsychiatric disturbances were added to the diagnostic criteria of AD that traditionally included cognitive and functional decline.^6,7^

Neuropsychiatric symptoms may co-occur, i.e., frequently several syndromes co-exist with another, such as depression with anxiety, depression with apathy, delusions with agitation, irritability with aggression, psychotic symptoms and wandering. Although correlated with one another, these disorders do not represent a single concept, and can be divided into distinct syndromic groups given their specific prevalence, clinical course, neurobiological bases, and psychosocial determinants.^8-10^

Unsurprisingly, diagnosis and treatment of these cooccurrences are a major challenge for clinicians.^11,12^ Although there is a worsening of neuropsychiatric symptoms with progressive impairment of cognitive decline in AD, this progression involving both processes does not always take place. Hence, in the mild phase of the disease, symptoms such as depression, anxiety, irritability and apathy are very common phenomena,^13^ while at advanced stages, the most common syndromes are aberrant vocalizations, delusions, hallucinations, wandering, agitation, aggression, besides persistent apathy.^14-16^

Although the steady progression of cognitive and functional decline remains the classic characteristic of AD, a worsening of neuropsychiatric symptoms following this deterioration is not always observed since depression and anxiety are commonly present among patients with mild disease, while aggression, agitation, and apathy occur more frequently in patients with severe disease.^17^

Another important issue concerns the onset of neuropsychiatric symptoms. These can occur in the prodromal phase of dementia, even in individuals without cognitive impairment and, when present, contribute to an increased risk of progression to dementia.^18,19^

Neuropsychiatric disturbances represent an important cause of patient suffering and early institutionalization, increase clinical comorbidities and the risk of mortality, while constituting great emotional stress and a heavy burden for family members and caregivers.^11,20,21^ Improving accuracy in diagnosing these syndromes represents a challenging concern for clinicians and researchers.

In addition to cognitive decline and functional deterioration, as well as changes on structural or functional neuroimaging and in CSF biomarkers, the diagnosis of AD currently encompasses neuropsychiatric symptoms at the early stages of dementia.^7,19,22^

Advances in the recognition and early accurate diagnosis of these syndromes helps guide appropriate pharmacological treatment and non-pharmacological interventions. The aims of this article were: [a] to discuss the main tools for evaluating behavioral disturbances, considered here as neuropsychiatric symptoms, commonly present in patients with dementia, particularly Alzheimer's disease; [b] to discuss the accuracy of neuropsychiatric scales, especially the Neuropsychiatric Inventory – Clinician Rating Scale (NPI-C).

## METHODS

For this work, methodological procedures were designed to critically discuss tools targeting the assessment of neuropsychiatric symptoms in patients with dementia, especially Alzheimer's disease. To this end, articles published on the PubMed database or in Brazilian journals were examined. The criteria for selected tools included available scales commonly used at Brazilian sites.

## Difficulties assessing neuropsychiatric syndromes in dementia.

The diagnosis of neuropsychiatric syndromes in dementia is a complex process that depends on the clinician's ability to distinguish psychopathological symptoms in the patient from the over or underestimated symptoms reported by the informant. In this scenario, emotional distress, caregiver burden, and cognitive impairment, often experienced by the caregiver, may affect the quality of their report.^5,23^

Therefore, depression, anxiety, sleep disorders, or irritability, among other symptoms, could interfere with the quality of the interpretation and description provided by the caregiver concerning psychopathological symptoms in the patient.^1,6^ For instance, the informant may undervalue an apathy picture since the patient apparently is quiet because of lack of motivation, reduced motor activities or less "distressing" behavior for others.^5,23^ Thus, a severe apathy condition tends not to be properly diagnosed when the report from the caregiver or family member is the main or sole reference available to the clinician.

The clinician rating approach attenuates misinterpretations from the caregiver's report in which their personal experience involving emotional distress, daily burden, or cognitive impairment may affect the ability to accurately describe the symptoms of the patient.^5,23^

The diagnosis of neuropsychiatric syndromes is crucial for pharmacological treatment or non-pharmacological intervention. Furthermore, the accurate assessment of symptoms remains an indispensable resource in studies and clinical trials. In this context, the diagnosis of psychopathological symptoms in dementia requires greater accuracy.^6,9^ Accordingly, determining a sound diagnosis is an indispensable task for the clinician to properly treat the symptoms.^6,24^ Thus, recommended standardized and validated measurements may contribute toward improving diagnosis for both scientific research and clinical practice.

## Assessments of neuropsychiatric symptoms in dementia.

There are several strategies for the assessment of neuropsychiatric symptoms in dementia. Brazilian centers attending elderly patients commonly use several scales in order to improve the recognition and measurement of psychopathological symptoms in dementia^3-5^ (Table 1).

**Table 1:** Available scales for use in Brazilian settings for assessing neuropsychiatric symptoms in dementia.

Author	Scale
Cummings et al., 1994	Neuropsychiatric Inventory (NPI)
de Medeiros et al., 2010	Neuropsychiatric Inventory – Clinician Rating Scale (NPI-C)*
Reisberg et al., 1996	Behavioral Pathology in Alzheimer's Disease scale (BEHAVE-AD)
Cohen-Mansfield, 1989	Cohen-Mansfield Agitation Index (CMAI)
Alexopoulos et al., 1988	Cornell Scale for Depression in Dementia (CSDD)
Robert et al., 2002; 2010	Apathy Inventory (AI)*
Reisberg et al., 1982	Global Deterioration Scale (GDS)

* Concurrent validity in Brazilian settings.

**Figure 1:**
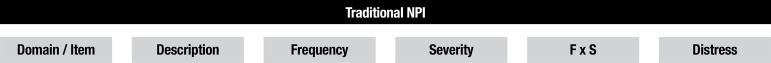
Structure of traditional Neuropsychiatric Inventory.

*The Neuropsychiatric Inventory (NPI) –* Among the standardized instruments available for measuring psychopathological symptoms of dementia, the Neuropsychiatric Inventory (NPI)^25^ is one of the most used tools. This scale comprises 12 domains targeting the identification of psychopathological symptoms in dementia. The clinician investigates each domain based on subjective description and scoring by the family member or caregiver regarding the patient's symptoms. The rater considers the frequency and intensity of symptoms in each domain. In addition, considering frequency and severity of symptoms, the informant reports the level of distress related to the groups of items within a given domain^25^ (Figure 1).

Although the NPI has been recognized by clinicians and researchers over the past two decades^10^ as the most applied scale for measuring neuropsychiatric symptoms in Alzheimer's disease and other dementias, this scale has some important shortcomings.^23^

Firstly, accurate scores for the frequency and severity of symptoms in the patient basically depends on the description of psychopathological symptoms by the caregiver in each domain. The information provided mainly by the caregiver may suffer interpretation bias. As mentioned above, daily burden and emotional distress, anxiety and depression, irritability and sleep disorders may interfere with the quality of information provided by the caregiver. In addition, the caregiver cannot sufficiently consider the clinical relevance of the symptoms when they are themselves elderly and may be affected by cognitive impairment.^5,23^

Furthermore, depending on regional and cultural features, the informant faces difficulty understanding specific symptoms present in the patient, such as apathy or depression.^5^ For instance, in several Brazilian groups, particularly among those who are illiterate, symptoms of apathy and depression may be considered "normal" characteristics of aging and not seen as relevant clinical syndromes.

Despite the indisputable contributions made by the traditional NPI, the problems mentioned above, including the absence of clinician scoring for each domain and the structure of this scale, represent the weakness of this tool.^23^

*Neuropsychiatric Inventory – Clinician Rating Scale (NPIC) –* With the participation of J.L. Cummings, responsible for creating the traditional NPI, de Medeiros et al.^23^ developed a new version of this scale called the *Neuropsychiatric Inventory – Clinician Rating Scale (NPIC)*. Thus, the NPI was restructured in a new version (the NPI-C), which includes the expansion of psychopathological items into multiple domains.

Furthermore, in the NPI-C, the domains *dysphoria, anxiety, elation, apathy, disinhibition, irritability, aberrant motor behavior, sleep disturbances* and *appetite and eating disorders* received new items.^23^ Only the domains *delusions* and *hallucinations* remained unchanged. In addition, the domain *agitation/aggression* from the traditional NPI was separated into two specific domains in the NPI-C. This division between *agitation* and *aggression* was based on studies of prevalence developed by Brodaty et al.^26^ These authors showed different frequencies for each domain. Accordingly, *agitation* and *aggression* present distinct psychopathological features and received new items. Patients with agitation do not exhibit verbal or physical behavior aimed directly at a specific target. However, these patients can manifest wandering behavior and uncooperative attitudes or resist care.^26^ Conversely, patients with aggression display behavioral disturbances as anger or attitudes against a particular target. Aggressive patients try to destroy objects or present intentional attempts to assault others, as well as themselves.^26^

A new domain (*aberrant vocalizations*) was incorporated into the NPI-C, which should be understood in the context of behavioral disturbances, being more common among patients with severe dementia. Therefore, 14 psychopathological domains, with inclusion of new items in each, composed the final version of the NPI-C. Moreover, the main feature that confers great strength to the NPI-C concerns its quality of accuracy.

**Figure 2:**
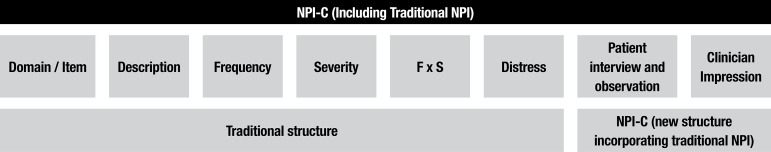
Structure of the Neuropsychiatric Inventory – Clinician Rating Scale (NPI-C).

Another important change concerns the scoring for each item and each domain performed by the clinician. As the rater decides on the severity for each item and each domain, the authors^23^ named this new tool the "Neuropsychiatric Inventory – Clinician Rating Scale" (NPI-C). Hence, the word "clinician" has been inserted in the title of the NPI-C. To improve the accuracy of assessment of symptomatology using the NPI-C, the clinician scores each item and each domain considering, in conjunction, several sources of information (Figure 2). Thus, clinicians also incorporate the report provided by the caregiver or family member, as well as data from the interview with the patient and direct observation of the patient's behaviors. If necessary, the clinician may seek additional data from the patient clinical records or may ask others for additional relevant clinical information.

To assess neuropsychiatric symptoms using the NPI-C, the clinician should consider psychopathological symptoms that the patient has presented over the past month. Finally, the clinician approach is part of the structure of the NPI-C. This feature provides good accuracy in recognizing the severity of neuropsychiatric symptoms in dementia and rectifies the weaknesses of the traditional NPI.^23^

Recently, in a multicenter cohort, we validated the NPI-C for the Brazilian setting, based on convergent validity and inter-rater reliability.^5^ To estimate the convergent validity of the NPI-C we correlated the 14 domains comprising this instrument with selected scales traditionally used to measure specific neuropsychiatric syndromes, namely: the Cohen-Mansfield Agitation Index,^27^ the Scale for Depression in Dementia,^28^ the Brief Psychiatric Rating Scale,^29^ and the Apathy Inventory,^30,31^ with high accuracy. Inter-rater reliability was also high. Currently, the NPI-C is available for use in Brazilian research and clinical settings.^5^

Summarizing, the NPI-C is a comprehensive and versatile approach for assessing neuropsychiatric symptoms in dementia with good accuracy and high reliability. This tool can be used as a comprehensive assessment of neuropsychiatric conditions or it can be applied to investigate specific psychopathological syndromes such as apathy, depression, delusions, hallucinations, agitation, sleep disorders, aberrant motor disorders, among others.^5,23^

*Behavioral Pathology in Alzheimer's Disease (BEHAVEAD) –* Reisberg et al.^17^ created the Behavioral Pathology in Alzheimer's Disease scale (BEHAVE-AD) aimed at assessing behavioral disturbances in AD patients, in addition to widespread cognitive approaches already in use across most centers. Another reason for devising this instrument was the need to measure behavioral changes in AD patients undergoing treatment with psychotropic drugs.^17^

The BEHAVE-AD consists of 25 items in which the family member or caregiver provides information about the level of severity of patient's behaviors for each question, over the past two weeks. This tool measures seven major categories of behavior disorders: paranoid and delusional ideation, hallucinations, disturbances of daily activities, aggression, sleep disturbances and circadian rhythm disorder, affective disorder, and anxiety or phobias. The score on each item ranges from 0 (absent) to 3 (present with intense emotional or physical involvement).^17^

Furthermore, through the BEHAVE-AD, it is possible to recognize whether behavioral disturbances are proving detrimental to the patient, as well as their impact on caregiver. The validity and reliability of this scale have been well established at research centers and clinical sites.

To avoid possible biases in interpretation by the caregiver, Auer et al.^32^ complemented the BEHAVE-AD with inclusion of direct observation of patient's behavioral disorders, and this tool was designated as the Empirical Behavioral Pathology in Alzheimer's Disease Rating Scale - E-BEHAVE-AD). This last instrument consists of 12 items entered under six categories of the BEHAVEAD, excepted for sleep disorders and circadian rhythm.^32^

*Cohen-Mansfield Agitation Index (CMAI) –* The purpose of the Cohen-Mansfield Agitation Index (CMAI)^27^ is the measuring of the frequency of agitated psychopathological behaviors in the elderly. Although originally developed to assess the efficacy of psychoactive drugs for agitation disturbances, especially among institutionalized populations, currently this inventory is being used in primary care centers and at other clinical sites for assessing neuropsychiatric symptoms of patients with dementia.

Based on the caregiver's report concerning the behavioral disturbances of the patient, the clinician completes the inventory. This scale comprises 29 items with scores ranging from 1 to 7 for each, according to the frequency of agitated behavior over the last two weeks. For scoring each item, the clinician should consider the progressive frequency of agitated behavior from 1 (never or almost never) to 7 (several times per hour). Some versions included an option to score 8 for behaviors that may occur when they cannot be avoided, besides a score of 9 for situations where the instrument is not applicable, for instance when the caregiver is unable to answer the questions or when the patient does not present agitation due to physical disability.^27^ In addition to the frequency of episodes related to agitated behaviors, later versions of the inventory included a description considering whether these symptoms cause difficulty for the patient and burden for the family, with scores ranging from 1 (never) to 5 (extremely). However, like in most scales, the clinician determines scoring on each item based on the subjective information from the caregiver.

*Cornell Scale for Depression in Dementia (CSDD) –* The Cornell Scale for Depression in Dementia (CSDD)^28^ is a tool originally developed to measure depressive symptoms in patients with dementia undergoing psycho-pharmacological treatment, especially antidepressants. The scale assesses the frequency and severity of depressive symptoms in demented patients over the last week. Accordingly, as in the majority of approaches, this instrument aims to quantify depressive symptoms, as well as provide support for clinical diagnosis.

Carthery-Goulart et al.^33^ translated the CSDD to the Brazilian Portuguese language with good reliability. This version remained stable in terms of its reproducibility in two situations: where the same rater administered the scale at different times; and, when different raters had applied it at the same time.

*Apathy Inventory (AI) –* Apathy is one of the most common neuropsychiatric conditions in some neurodegenerative diseases such as Alzheimer's disease and Parkinson's disease. Apathy also can co-occur with depression, despite having a different clinical courses and with distinct neuropathological correlates involved in each syndrome.^34^ Several studies have reported a high prevalence of apathy in patients with Alzheimer's disease, occurring in 55-70% of cases.^10,23,35^

This syndrome clinically comprises reduced or absence of motivation in three apathy dimensions - emotional reactivity, cognitive interest, and behavioral initiative. The Apathy Inventory (AI)^30,31^ is an efficacious tool for assessing the three mentioned dimensions. Thus, this inventory comprises items related to decreased motivation originating from internal or external stimuli with: [a] reduction or loss of affective and emotional reactivity; [b] reduction or loss of cognitive interest or goal-directed cognitive activity; and [c] reduction or loss of initiative or goal-directed behavior.

One methodological quality of the AI is its high level of accuracy. Similarly to the NPI-C, the scoring of the AI requires the responses from the caregiver or family member, but the clinician ultimately decides the score for each item and each domain. Therefore, to score the AI, the clinician considers reports from the caregiver or family member, but determines a severity rating for each item and each domain. Furthermore, the clinician establishes the final score based on the interview with the patient and takes into account the behavioral reactions during the evaluation.

Patients with dementia and apathy commonly do not cause "disturbances" to others. Because of this attribute, sometimes the caregiver cannot recognize apathetic symptoms. Despite the high frequency and clinical relevance of apathy, caregivers have a tendency to overlook the patient's suffering.^5,23^

Moreover, caregivers often experience depression, sleep disorders, or when elderly, also present cognitive decline. In addition to the daily burden, these psychiatric conditions can lead caregivers to overvaluing the severity of symptoms or misidentifying the presence of the syndrome in demented patients.^23,36^ In this scenario, the decision by the clinician regarding the three dimensions of apathy remains a critical strategy to appropriately recognize and measure the symptoms, and this approach confers greater accuracy for assessments of the syndrome severity.^30,31^

Recently, we validated the AI for Brazilian settings, and this tool is available for assessment of apathetic symptoms in patients with Alzheimer's disease, Parkinson's disease, depression, and mild cognitive impairment.^36^ The authors found strong internal consistency of the tool, a good level of concurrent validity (with the apathy domain from the NPI-C), as well as high inter-rater reliability. Furthermore, the study established a high sensitivity and specificity of the AI. This study revealed that this tool is an available and easy approach to assess apathy syndrome across several neuropsychiatric conditions.

Other scales have been used to assess apathy symptoms in elderly patients. Several groups have applied the Apathy Scale^37^ or the Apathy Evaluation Scale^38^ to assess symptomatic manifestations in patients with neurodegenerative processes, such as Alzheimer's disease and Parkinson's disease. However, there is a weakness of these instruments regarding the scoring for each item in that this relies on a report from the caregiver, not requiring the clinician's impression.

Finally, the apathy domain from the NPI-C (NPI-C/Apathy) could be used to assess apathetic symptoms, with the advantage that the clinician determines the scoring for each item based on their clinical impression, as also seen in the Apathy Inventory.

*Global Deterioration Scale (GDS) –* The Global Deterioration Scale (GDS)^39^ was developed to measure cognitive decline and its impact on the overall functioning of patients with dementia in neurodegenerative conditions, particularly Alzheimer's disease. This scale comprises seven different levels of severity related to the progressive global deterioration of the patient. Level 1 refers to the absence of cognitive decline. Levels 2 and 3 denote, very mild and mild cognitive decline, respectively. The subsequent levels 4-7 include stages of clear overall deterioration, reaching severe cognitive and functional decline.^39^ Although the GDS has not been designed primarily to investigate behavioral disorders, it can be used to identify some neuropsychiatric disturbances, such as aggressiveness, agitation, and disorganized behavior, particularly in advanced stages of dementia.

*Other scales –* Other scales have also been used with the purpose of measuring behavioral disturbances in patients with dementia.

Overall and Gorhan^40^ created the Brief Psychiatric Rating Scale (BPRS) to measure changes in psychopathological severity of patients with chronic psychotic conditions, such as schizophrenia, or persistent delusional disorder. However, several groups have applied this scale to assess psychotic symptoms or disorganized behaviors in patients with dementia. The version composed of 18 items has been the most used and includes a class of symptoms that are relevant for clinical approaches including delusions, hallucinations, and disturbed behaviors. The scale has good reliability for the items responsible for identifying key psychotic symptoms. Romano and Elkis^41^ translated and adapted a specific form of this tool called the anchored version (BPRS-A), for use in the Brazilian community.

Another instrument, known as the Sandoz Clinical Assessment-Geriatric Scale,^42^ allows the assessment of cognitive changes, as well as irritability, hostility, anxiety, and depressive symptoms. However, this scale has been less used in Brazilian settings.

**Final comments.** In recent years, the diagnosis of dementia, including Alzheimer's disease, has been improved by combining neuropsychiatric symptoms with cognitive decline and functional deterioration, traditionally adopted as clinical criteria.^6,7,11,22,24^

The accuracy of symptom assessment is essential for sound diagnosis of psychopathological syndrome, and thus for treatment. However, the caregiver or family member may suffer from emotional distress and excessive daily burden, or potential cognitive decline when elderly. Taken together, these factors could lead to biases in recognizing and interpreting the symptoms of the patient.^5,23^

In this scenario, the role of the clinician remains crucial to establish the final diagnostic impression regarding neuropsychiatric symptoms.^5,23^ Clearly, the information provided by the family member or caregiver is important and should be considered accordingly. Moreover, the clinician should actually interview the patient, directly observe patient reactions during the assessment, and access other clinically relevant and available data from medical records in order to improve the accuracy of the evaluation.^5,23^

In agreement with consensus recommendations for the diagnosis of AD recently published,^43^ it is plausible to strongly consider behavioral disturbances as part of the clinical manifestations of the disease. In addition to selected tools aimed at assessing neuropsychiatric symptoms, this approach may improve diagnostic accuracy.

Currently, there are several instruments validated for use in our milieu, and these are available to clinicians and researchers for assessing psychopathological manifestations in patients with dementia, including Alzheimer's disease and other conditions characterized by cognitive and functional decline.

The traditional NPI represents the most widely used scale for the assessment of neuropsychiatric symptoms in dementia. Although this tool has contributed significantly toward evaluating behavioral disturbances in Alzheimer and other dementias, a major weakness of this approach concerns the absence of clinician scoring for each domain.^23^ Caregivers frequently suffer emotional distress and excessive burden, as well as cognitive decline when elders. Taken together, these factors hinder the caregiver in interpreting and describing neuropsychiatric symptoms of the patient.^23,24,44^

Also validated in a Brazilian cohort, the NPI-C represents a valuable tool for use across clinical trials or research centers to assess neuropsychiatric symptoms of dementia, offering good accuracy and reliability.^5^ As ultimately the clinician decides on the severity of the symptoms in patients with dementia, the accuracy of the NPI-C domains is rendered more reliable.^5,23^
